# Hypernatremic dehydration, diabetes insipidus, and cerebral venous sinus thrombosis in a neonate: a case report

**DOI:** 10.1186/1752-1947-1-66

**Published:** 2007-08-21

**Authors:** Laurene M Fleischer, Thomas A Wilson, Margaret M Parker

**Affiliations:** 1 Department of Pediatrics, Stony Brook University Medical Center, Stony Brook, NY 11794-8111, USA

## Background

In breast-fed neonates, hypernatremia may occur from inadequate breast milk production, central or nephrogenic diabetes insipidus, gastroenteritis, or salt poisoning. Hypernatremic dehydration is a known risk factor for cerebral sinus thrombosis and death. We report a breastfed neonate with hypernatremic dehydration, diabetes insipidus, cerebral sinus thrombosis, and disseminated intravascular coagulation. We discuss the difficulties in determining cause and effect, as well as emphasize the importance of early evaluation of excessive weight loss in the neonate.

## Case presentation

A 13 day old Caucasian infant presented with failure to thrive, dehydration, and listlessness. She was born at 37 weeks following induced labor and vacuum-assisted vaginal delivery because of oligohydramnios. Birth weight was 2.9 kg. The mother had been treated with metformin for polycystic ovary syndrome until the 2nd trimester, terbutaline for asthma, and amoxicillin for a urinary tract infection. The baby was exclusively breastfed. On day 10 she was seen by her primary care physician because of poor feeding but was noted to have good urine output (about 5–6 wet diapers per day according to her parents). Her weight was 1.9 kg. Supplemental feedings with formula were recommended. Feedings remained poor and on day 13 she was admitted to our institution after presenting to an outside hospital with lethargy and dehydration. Admission weight was 2.3 kg (on a different scale), temperature of 98°F rectally, heart rate 125, respiratory rate 32, and blood pressure 106/72. She appeared alert but cachectic, had a sunken anterior fontanel, dry mucous membranes, capillary refill of 4 seconds, and mild tenting of the skin. Serum sodium level was 173 mmol/L, potassium was unavailable due to hemolysis, chloride 140 mmol/L, bicarbonate 20 mmol/L, blood urea nitrogen 143 mg/dL, creatinine 1.6 mg/dL, glucose 120 mg/dL, calcium 10.5 mg/dL. Initial CBC revealed a white blood cell count of 16.8, hemoglobin 18.9, hematocrit 56.3, likely due to hemoconcentration. On follow up CBC 12 hours later the white blood cell count was 9.9, hemoglobin 14.0, and hematocrit 41.3.

The patient received two normal saline boluses at 20 cc/kg intravenously, then 0.25% saline at maintenance on presentation to the outside hospital prior to the results of her chemistry panel. Urine output remained brisk, at 1 cc/kg/hr for the first 5 hours even though the patient was clinically dehydrated. A foley catheter was placed for more accurate monitoring and her intravenous fluids were adjusted according to her electrolytes which were checked every 6 hours. Urine output over the first day of admission increased to 4.7 cc/kg/hr. The patient became more lethargic, had persistent hypernatremia, and developed respiratory distress and intermittent bradycardia. Her right pupil became fixed and dilated and she was intubated. A head ultrasound suggested an intracranial bleed on the right side, and a subsequent head CT showed large parenchymal hemorrhages in the right frontal lobe, with marked cerebral edema and a midline shift. She developed seizures. Coagulation studies revealed a platelet count of 20,000/ul, prothrombin time greater than 120 sec, and partial thromboplastin time greater than 240 sec. Her fibrinogen was less than 58 mg/dL (normals:150–400 mg/dl), the D dimer was 2912 ng/mL (normal < 200 ng/ml). She received transfusions of platelets, fresh frozen plasma, and cryoprecipitate, with correction of her coagulopathy.

Seizure activity increased, she developed hypotension, and both pupils became dilated and non-reactive. CT scan showed markedly increased cerebral edema, with loss of gray-white matter differentiation. Anticonvulsants and dopamine were started. While on IV fluids, she continued to have a high urine output, with serum osmolality of 330 mosm/kg, urine osmolality of 248 mosm/kg, high serum Na, low urine Na, and low urine specific gravity (Table 1). Two hours after receiving one dose of desmopressin (DDAVP) subcutaneously (0.002 micrograms/kg), her urine output decreased to 2.9 cc/kg/hr, serum osmolality decreased slightly, while urine osmolality, urine Na, and urine specific gravity all increased (Table 1). She became hemodynamically stable and her electrolytes improved, but her neurological status continued to deteriorate. She became flaccid with no cranial nerve activity and had no spontaneous respiratory effort. Mechanical ventilation was withdrawn. An autopsy showed central venous sinus thrombosis of the transverse and superior sagittal sinuses with subarachnoid and intraparenchymal hemorrhage and severe cerebral edema. Because of extensive liquefaction necrosis, the posterior pituitary gland could not be identified. The anterior pituitary gland appeared structurally normal.

**Table 1:** Serum and urine laboratory values before and after administration of desmopressin (DDAVP).

	Admission	12 Hours After Admission	Day 3, Before DDAVP	Day 3, 4 hrs After DDAVP
**Serum**:				
Na	173 mmol/L	157 mmol/L	163 mmol/L	154 mmol/L
CO2	20 mmol/L	22 mmol/L		24 mmol/L
BUN	143 mg/dL	80 mg/dL		19 mg/dL
Creat	1.6 mg/dL	0.9 mg/dL		0.6 mg/dL
osmolality			330 mosm/kg	324 mosm/kg
**Urine:**				
Output			6.5 cc/kg/hr	2.9 cc/kg/hr
Spec.grav.			1.007	1.012
Osmolality			248 mosm/kg	413 mosm/kg
Na			57 mmol/L	125 mmol/L

## Discussion

Hypernatremic dehydration in exclusively breast fed infants is uncommon, but well documented [1,2]. A prospective study by Manganaro et al, found that 7.7% of term, healthy neonates being exclusively breastfed had a weight loss greater than 10% in the first week of life. Thirty six percent of those with weight loss exceeding 10% were hypernatremic, with a maximum sodium concentration of 160 mmol/L, which returned to normal with adequate hydration [1]. Most often weight loss and hypernatremic dehydration can be explained by inadequate volume of intake secondary to insufficient maternal milk production or poor breastfeeding technique. There is some evidence to support the theory that increased sodium in the breast milk also plays a role [3]. The present case is unique due to the diagnosis of central diabetes insipidus (DI) and central venous sinus thrombosis. A major issue is whether the DI was the cause of her hypernatremic dehydration or resulted subsequent to the central venous sinus thrombosis.

Certain important points in this case argue for DI as being the initial diagnosis leading to the hypernatremic dehydration. Despite poor intake and very significant weight loss prior to admission, the baby continued to have 5–6 wet diapers per day. In addition, although she did not have true polyuria on admission, 1 cc/kg/hr of urine output in the setting of severe dehydration is excessive. Unfortunately, the initial urinalysis was not performed until approximately 16 hours after the patient was admitted, at which time the specific gravity was 1.010, which cannot be interpreted as the patient had already received several hours of intravenous fluids. However, the subsequent laboratory values (see Table 1), and response to DDAVP support the diagnosis of DI. The history of oligohydramnios, as opposed to polyhydramnios as is classically seen with DI in utero, suggests that DI developed postnatally in this infant. Brown and Alward observed central DI in a series of 3 neonates born through complicated labors and deliveries. These patients manifested symptoms within the first 72 hours of life [4]. Smith and Friden described central DI in a 21 day old neonate who was subsequently diagnosed with septo-optic dysplasia [5].

On the other hand, central DI can be a complication of intracranial hemorrhage, cerebral infarct, or DIC [6,7] and dural sinus thrombosis has been reported as a complication of hypernatremic dehydration in a breastfed neonate [8]. The diagnosis of DI was not confirmed until after this child had developed the central venous thrombosis even though she had many abnormalities suggestive of DI on admission. It was impossible to determine the sequence of events conclusively by autopsy. In addition, despite our efforts to slowly correct the patient's hypernatremia, her sodium did decrease from 173 to 157 within 12 hours. It is possible that this rapid correction could have caused some cerebral edema contributing to her neurological deterioration.

In a review of the current literature on hypernatremic dehydration in breastfed infants, van Amerongen et al cited the most common complications are neurologic, including seizures, cerebral edema, cerebral vascular accidents, paralysis, and encephalopathy [2]. The second most common complication was disseminated intravascular coagulation (DIC). This patient exhibited both neurologic complications (seizures, cerebral edema) and DIC. We postulate that the hypernatremic dehydration caused a cerebral sinovenous thrombosis, which then led to a consumptive coagulopathy and secondary widespread intracranial hemorrhage. The most common presentation of sinus thrombosis in neonates is seizures [9]. Van Amerongen et al described a case with a course similar to that of our patient in which a 14 day old breastfed infant presented with severe hypernatremic dehydration, with a serum sodium of 213 mmol/L, who then developed apnea at 48 hours after admission. The patient then progressed to have a bulging anterior fontanel, fixed and dilated pupils, and hypotonia. Head CT revealed cerebral edema and transverse venous sinus thrombosis [2].

The Canadian Pediatric Ischemic Stroke Registry studied sinovenous thrombosis in 160 consecutive children [9]. Among 104 children who underwent both CT and MRI, CT did not detect the sinovenous thrombosis in 16%. Wu et al advocate that, "any full term infant with unexplained hemorrhage into the ventricles or in the deep gray structures of the brain should be imaged with MRI and MR venography to rule out a sinovenous thrombosis" [10]. In the present case, CT did not demonstrate venous sinus thrombosis, but did show extensive parenchymal hemorrhage and edema. Venous sinus thrombosis was apparent only on autopsy. An MRI may have demonstrated the venous sinus thrombosis, but was not done because of the critical clinical status of the patient. Also, due to the coagulopathy and bleeding, anticoagulant therapy for the venous sinus thrombosis was not an option.

## Conclusion

Close follow up of infants during the first days after hospital discharge, particularly in breast fed infants, is essential for the primary care physician to detect early weight loss. Even in the infant who looks well, weight loss of 10% or more deserves immediate investigation, including evaluation of the serum electrolytes for possible hypernatremia. It has been shown that prompt diagnosis and treatment of weight loss due to hypernatremic dehydration results in good outcome [1].

Urine output is not a good indicator of hydration status in children with DI and can lead to inappropriate reassurance. In addition, patients with hypernatremic dehydration may clinically appear relatively well, and therefore laboratory investigation is necessary to make the diagnosis. Once recognized, hypernatremic dehydration in the neonate must be evaluated expeditiously to investigate the possibility of DI and other causes. It is important to check paired serum and urine osmolarity, urine specific gravity and urine electrolytes. The combination of hypernatremia, inappropriately low urine specific gravity and low urinary sodium concentration will confirm a diagnosis of diabetes insipidus. A simultaneous measurement of serum ADH or the response to ADH will indicate whether the DI is central or nephrogenic. Appropriate management, including prompt treatment with DDAVP, will optimize outcome which otherwise can be catastrophic.

## Competing interests

The author(s) declare that they have no competing interests.

## Authors' contributions

All of the authors listed have contributed to the writing of this manuscript, have been involved in subsequent revisions, and have given final approval of the version to be published.
